# Effects of *Lactobacillus casei* Shirota ingestion on common cold infection and herpes virus antibodies in endurance athletes: a placebo-controlled, randomized trial

**DOI:** 10.1007/s00421-016-3415-x

**Published:** 2016-06-13

**Authors:** Michael Gleeson, Nicolette C. Bishop, Lauren Struszczak

**Affiliations:** School of Sport, Exercise and Health Sciences, Loughborough University, Loughborough, Leicestershire LE12 3TU UK

**Keywords:** Exercise training, Herpesvirus, Probiotic, Common cold

## Abstract

**Aims:**

To assess evidence of health and immune benefit by consumption of a *Lactobacillus casei* Shirota probiotic in highly physically active people.

**Methods:**

Single-centre, population-based, randomized, double-blind, placebo-controlled trial. Daily ingestion of probiotic (PRO) or placebo (PLA) for 20 weeks for *n* = 243 (126 PRO, 117 PLA) university athletes and games players. Subjects completed validated questionnaires on upper respiratory tract infection symptoms (URS) on a daily basis and on physical activity status at weekly intervals during the intervention period. Blood samples were collected before and after 20 weeks of the intervention for determination of Epstein Barr virus (EBV) and cytomegalovirus (CMV) serostatus and antibody levels.

**Results:**

URS episode incidence was unexpectedly low (mean 0.6 per individual) and was not significantly different on PRO compared with PLA. URS episode duration and severity were also not influenced by PRO. A significant time × group interaction effect was observed for plasma CMV antibody titres in CMV seropositive participants (*p* < 0.01) with antibody titre falling in the PRO group but remaining unchanged in the PLA group over time. A similar effect was found for plasma EBV antibody titres in EBV seropositive participants (*p* < 0.01) with antibody titre falling in the PRO group but increasing in the PLA group over time.

**Conclusions:**

In summary, regular ingestion of PRO did not reduce URS episode incidence which might be attributable to the low URS incidence in this study. Regular ingestion of PRO reduced plasma CMV and EBV antibody titres, an effect that can be interpreted as a benefit to overall immune status.

## Introduction

Probiotics are food supplements that contain “friendly” gut bacteria. There is now a reasonable body of evidence that regular consumption of probiotics can modify the population of the gut microflora and influence immune function (Trejo and Sanz 2013).

Many studies have been conducted on the effects of probiotic use on upper respiratory tract infection symptom (URS) episodes in the general population and a recent systematic review (King et al. 2014) concluded that probiotic use resulted in fewer numbers of illness days, shorter illness episodes and fewer days absence from day care/school/work. The most recent Cochrane systematic review of probiotic benefits for URS using data from randomised controlled trials involving 3720 non-athletes concluded that probiotics were better than placebo in reducing URS episode incidence by about 47 %, and the average duration of an acute URS episode by about 2 days (Hao et al. 2015).

Prolonged intense exercise has been associated with a transient depression of immune function (Walsh et al. 2011a; Gleeson et al. 2013b) and a heavy schedule of training and competition can lead to immune impairment in athletes, associated with an increased susceptibility to upper respiratory tract infection symptoms (URS) (Walsh et al. 2011a; Gleeson et al. 2013a, b). Both aspects of innate immunity (e.g., neutrophil chemotaxis, phagocytosis, degranulation and oxidative burst activity, monocyte toll-like receptor expression and natural killer cell cytoxic activity) and acquired immunity (e.g., antigen presentation by monocytes/macrophages; T lymphocyte cytokine production and proliferation, immunoglobulin production by B lymphocytes) are depressed by prolonged exercise (Walsh et al. 2011a; Gleeson et al. 2013b). The causes of immune depression after prolonged exercise are thought to be related to increases in circulating stress hormones (e.g., adrenaline and cortisol), alterations in the pro-/anti-inflammatory cytokine balance and increased oxidative stress. To date there are relatively few published studies of the effectiveness of probiotic use in athletes and games players; a recent comprehensive review by Pyne et al. (2015) identified 15 relevant experimental studies that investigated immunomodulatory and/or clinical outcomes. Of those studies that recorded URS episode incidence, five found reduced URS frequency or fewer days of illness (Cox et al. 2010; Gleeson et al. 2011; West et al. 2011, 2014; Haywood et al. 2014), and three reported trivial or no effects (Kekkonen et al. 2007; Tiollier et al. 2007; Gleeson et al. 2012). More recently, another published study on athletes reported a reduction in the duration, number and severity of URS compared with placebo although URS episode incidence was not affected (Marinkovic et al. 2016a). Studies that examined immunomodulatory effects of probiotics in athletes have reported increased interferon-γ production in whole blood culture (Clancy et al. 2006) and T-cells (Cox et al. 2010) and better maintenance of salivary SIgA during intensive training (Tiollier et al. 2007; Gleeson et al. 2011; Marinkovic et al. 2016b).

An additional measure that can be used to establish if regular probiotic has an impact on overall immune status is the change in the plasma antibody levels to the herpes viruses, cytomegalovirus (CMV) and Epstein Barr virus (EBV), from the start to the end of the study. The level of CMV-specific immunoglobulin G (IgG) in plasma of CMV seropositive individuals inversely reflects the overall immune system status and is part of the immune risk profile used to assess immune dysregulation in elderly people (Hadrup et al. 2006; Alonso Arias et al. 2013). A recent double blind, placebo controlled study using a daily *Lactobacillus* probiotic for 6 months reported that the serum CMV IgG titre was significantly increased at the end of the study in the placebo group but was unchanged in the probiotic group possibly because their immune system was able to better control viral reactivations (Moro-García et al. 2013). Similarly, the change in the plasma Epstein Barr virus (EBV)-specific immunoglobulin G (IgG) titre from the start to the end of the study could also be used to assess any change in overall immune status. Previous studies have established that stress-related immunosuppression can significantly increase EBV and/or CMV antibody titres (Glaser et al. 1985, 1999; Godbout and Glaser 2006). Conversely, improved cell-mediated immunity is indicated when an intervention causes levels of these antibodies to fall (Dowd et al. 2011; Moro-García et al. 2013).

Hence, the aims of the present study were to examine the effects of 5 months of daily oral supplementation of a fermented milk drink containing the gram positive probiotic *Lactobacillus casei* Shirota, on URS episode incidence, the duration and severity of episodes, and plasma CMV and EBV antibody titres in a cohort of university-based endurance athletes and games players. We chose to examine this particular probiotic because it is one of the most popular commercially available probiotics with world-wide availability and there is strong evidence that the bacteria it contains survive gastrointestinal transit and modify the gut microbiota population (Matsumoto et al. 2006; Spanhaak et al. 1998) and can alter some aspects of systemic immunity in humans (Dong et al. 2010, 2012; Matsuzaki, 1998; Nagao et al. 2000).

## Methods

### Subjects

Two hundred and sixty-eight subjects (156 males and 112 females) who were engaged in regular sports training (predominantly endurance-based activities such as running, cycling, swimming, triathlon, and team games) volunteered to participate in the study. Subjects ranged from recreationally active to national level athletes and their self-reported training loads averaged 11 h/week. Subjects were required to complete a comprehensive health-screening questionnaire prior to starting the study and had not taken any regular medication, antibiotics or probiotics in the 3 months prior to the study. All subjects were fully informed about the rationale for the study and of all experimental procedures to be undertaken. Subjects provided written consent to participate in the study, which had earlier received the approval of Loughborough University ethical advisory committee. Subjects were enrolled after having fulfilled all inclusion criteria, and presenting none of the exclusion criteria (determined by both questionnaire and interview).

Subjects could be included if they were currently healthy, had been involved in endurance training for at least 2 years, engaged in at least three sessions and at least 3 h of total moderate/high-intensity training time per week and were between 18–50 years of age. Subjects representing one or more of the following criteria were excluded from participation: smoking or use of any medication, currently taking probiotic supplements, suffered from or had a history of cardiac, hepatic, renal, pulmonary neurological, gastrointestinal, haematological or psychiatric illness; objected to the prescription of diet (abstinence from fermented milk products other than the daily supplement).

Subjects were randomly assigned to one of two treatments (probiotic or placebo) with stratification by gender and type of sport (A: endurance sports such as triathlon, swimming, cycling and distance running, *n* = 66; B: individual sports such as tennis, squash and badminton, *n* = 33; C: team games such as football, rugby, hockey, lacrosse and basketball, *n* = 169). All subjects were based on campus except for five athletes who were from local non-university sports clubs. Under double-blind procedures 137 received the probiotic and 131 received the placebo preparation. Of these 268 subjects 113 (42 %) were female and 155 (58 %) were male. Their ages ranged from 18 to 32 with the mean age of the study cohort at recruitment being 21 ± 3 years (mean ± SD).

### Study intervention

Probiotic (PRO) and placebo (PLA) supplements were supplied as fermented milk in sealed pots of 65 ml with date stamped expiry. The PRO drink contained a minimum of 6.5 × 10^9^ live cells of *Lactobacillus casei* Shirota in each pot. The PLA was identical in taste and colour to the PRO but contained no lactobacilli. The supplements were stored at 3–5 °C and a fresh supply was provided by the manufacturer (Yakult Europe, Amsterdam, The Netherlands) every 2 weeks. Subjects returned to the laboratory every 2–3 weeks to receive a fresh supply of supplement. This was provided in a cardboard box labelled with one of six letters (N, Q, R, S, T, or U). Three of these letters (N, T, U) corresponded to PRO and the other three (Q, R, S) to PLA (blinded to both the subjects and the investigators). A compliance log of sample collection was taken. Subjects consumed the supplement twice per day; one 65 mL pot with breakfast and one with the evening meal for 20 weeks. Subjects were asked to keep a record of any days when they missed taking the supplement.

### Study protocol

For the first visit to the laboratory, subjects arrived in the morning at 08:30–10:30 following an overnight fast of approximately 12 h and their body mass and height were recorded. Information about the study had previously been given to them and they signed an informed consent form. Subjects then sat quietly for 10 min and completed a health-screening questionnaire and inclusion/exclusion criteria questionnaire. A resting venous blood sample (5 ml) was obtained by venepuncture from an antecubital forearm vein into a Vacutainer tube (Becton–Dickinson, Oxford, UK) containing K_3_EDTA. Haematological analysis was immediately carried out on this sample (including haemoglobin, haematocrit and total and differential leukocyte count) using an automated cell-counter (A^c^.T™5diff haematology analyser, Beckman Coulter, High Wycombe, UK). Subjects had to have normal haematology to be included in the study. The remaining blood was centrifuged for 10 min at 5000*g* and 4 °C and the plasma stored at −80 °C prior to analysis. Subjects eligible for inclusion in the study were randomly assigned to the treatment or placebo group and asked to start taking the supplement the next day.

During the 20-week intervention period with PRO or PLA subjects were requested to continue with their normal training programs. Other supplements (Gleeson 2016) that might influence immune function (e.g. vitamins, minerals, colostrum, flavonoids) and consumption of additional probiotics or any fermented dairy products (e.g. yoghurt, sour cream, crème fraiche) were not permitted during this period. Subjects completed a validated URS questionnaire (Jackson et al. 1958) on a daily basis. Subjects were not required to abstain from medication when they were suffering from illness symptoms but they were required, on a weekly basis, to report any unprescribed medications taken, visits to the doctor or any prescribed medications.

The illness symptoms listed on the questionnaire were: sneezing, headache, malaise, nasal discharge, nasal obstruction, sore throat, cough, ear ache, hoarseness, fever, chilliness and joint aches and pains. The non-numerical severity ratings of mild, moderate and severe of severity of symptoms were scored as 1, 2 or 3, respectively to provide a quantitative means of data analysis and the total symptom score for every subject each day was calculated as a sum of multiplied numbers of symptoms experienced by the numerical severity ratings. An URS (common cold) episode was deemed present when both (1) total symptom score was ≥15 on any two consecutive days and (2) when a subject positively indicated suffering a common cold on ≥3 days (Jackson et al. 1958). Subjects were also asked to rate the impact of illness symptoms on their ability to train (above normal, at the same level, below normal or training stopped). The total number of URS days was also determined as the number of days with a symptom score of ≥5 according to Predy et al. (2005).

Subjects were also asked to fill in a standard short form of International Physical Activity Questionnaire (IPAQ; http://www.ipaq.ki.se/downloads.htm) at weekly intervals, thus providing a quantitative information on training loads in metabolic equivalents (MET)-h/week (Craig et al. 2003). Subjects attended the laboratory every 4 weeks for collection of fresh product and recording of body mass. In addition, venous blood samples were collected during the final visit.

### Plasma analysis

Thawed plasma samples were analysed for CMV serostatus and CMV-IgG titre by enzyme-linked immunosorbent assay (ELISA) (CMV IgG ELISA kit GD84, Genesis Diagnostics, Littleport, Cambridgeshire, UK supplied by Biohit Healthcare Ltd, Ellesmere Port, Cheshire, UK). Samples with an absorbance less than that of the 3 IU/mL standard were considered negative for anti-CMV IgG. In CMV seropositive samples, antibody titre was determined by interpolation from a calibration curve using standards of 0, 3, 10 and 30 IU/mL provided with the kit. Plasma samples were also analysed for EBV serostatus and EBV-IgG titre by ELISA (Novagnost EBV-VCA IgG ELISA kit, NovaTec Immunodiagnostica GmbH, Dietzenbach, Germany, supplied by Siemens Healthcare Diagnostics Ltd, Camberley, Surrey, UK). Samples with an absorbance value higher than 15 % over the cut-off standard (10 U/mL) were considered positive for anti-EBV IgG. In EBV seropositive samples, antibody titre was determined by calculation of the increase above that of the cut-off standard assuming a linear calibration curve as recommended by the manufacturer.

### Statistical analysis

Sample size estimation of at least 96 subjects per treatment group was determined using G*Power 3.1 (Faul et al. 2009) based on an expected rate of 2.1 ± 1.2 URS episodes (mean ± SD) during 4 winter months (Gleeson et al. 2011), a target 20 % reduction in number of episodes (to 1.7 ± 1.0 URS episodes), statistical power of 80 % and type I error of 5 %. The outcome revealed that a total of 192 participants (per 2 arms) was required. We initially recruited 268 volunteers to account for an anticipated ~30 % drop-out rate over the study period. Any difference in the proportion of subjects who presented with one or more episodes of URS during the trial between the PRO and PLA groups was assessed by a Chi squared test. Comparison between the treatments for the number of URS episodes was carried out using a Mann–Whitney test. For subjects who experienced one or more URS episodes, comparison between the treatments for total symptom severity score and the mean duration of URS episodes was carried out using unpaired t tests. Comparison between the treatments for the number of days with an URS score ≥5 (Predy et al. 2005) was carried out using a Mann–Whitney test. Changes in plasma CMV and EBV antibody titres in seropositive participants before and after 20 weeks of the treatment period were analysed using a between (treatment) within (time) two factor repeated measures ANOVA. When Mauchly’s test of sphericity was significant, the Greenhouse-Geisser correction to the *F* values was applied. When significant main effects were found the Bonferroni post hoc test was applied to locate the differences. Data are presented as mean (±SEM). The accepted level of significance was *p* < 0.05.

## Results

### Adherence to the study

Of the 268 subjects, 137 were allocated to PRO and 131 to PLA. There should, of course, have been equal numbers on PRO and PLA. We identified that during the first visit to the lab and treatment allocation five participants (one cyclist and four swimmers) were wrongly coded as individual sports (Group B) when they should have been coded as endurance athletes (Group A). Retrospective analysis following treatment unblinding revealed that for three of these participants this error made no difference to their treatment allocation but resulted in the two of them being allocated to PRO rather than PLA. A total of 243 subjects (126 PRO, 117 PLA) completed the full 20 weeks of the study and the proportion of males (0.58) was the same in both PRO and PLA groups. The number of dropouts was smaller than anticipated and there were slightly fewer (*n* = 11) dropouts on PRO than on PLA (*n* = 14). Reasons for dropout included going abroad on vacation during the Christmas period (*n* = 1), leaving the university (*n* = 5), injury (*n* = 4), persistent non-respiratory illness preventing them from performing training (*n* = 2), disliking the product (*n* = 5) or due to undisclosed reasons (*n* = 8).

Adherence to the intervention was good: subjects who completed the study reported that they missed taking the supplement on average only on 4 days. Blood samples were obtained pre- and post-intervention from 223 subjects.

### Patterns in baseline characteristics

Baseline characteristics of the subjects who completed the study, including body mass, height, body mass index (BMI) and self-reported weekly training duration for the PRO and PLA groups are shown in Table 1. The baseline values were similar with no statistically significant difference between the two treatment groups. By the end of the study body mass had increased by 0.7 ± 0.2 kg (*p* < 0.001) with the increase for participants on PRO (0.9 ± 0.2 kg) being slightly higher but not significantly different (*p* = 0.564) than that for PLA (0.5 ± 0.2 kg).

**Table 1:** Baseline characteristics of the subjects who completed the study

	Probiotic	Placebo
Number (M, F)	126 (73, 53)	117 (69, 48)
Sport (A, B, C)	33, 16, 77	31, 14, 72
Age (years)	20.3 ± 0.2	20.6 ± 0.2
Body mass (kg)	72.3 ± 1.1	73.3 ± 1.0
Height (cm)	176.0 ± 0.9	176.6 ± 0.9
BMI (kg/m^2^)	23.2 ± 0.2	23.4 ± 0.2
Training (h/week)	11 ± 1	11 ± 1

Values are expressed as mean ± SEM. No significant differences between PRO and PLA

*M* males, *F* females, *A* endurance sports, *B* individual sports, *C* games players

### Physical activity levels

**Fig. 1:**
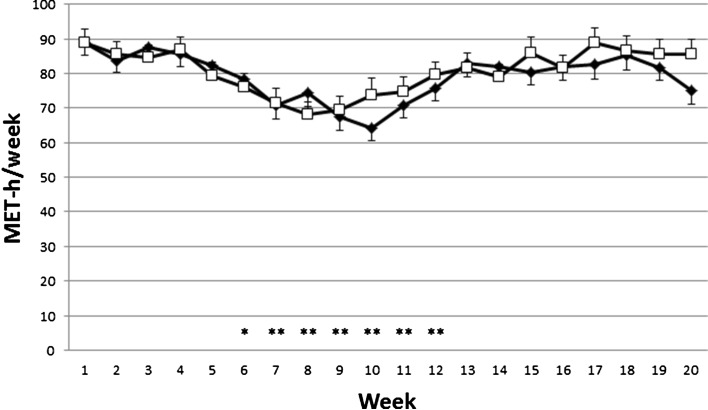
Training loads in MET-h/week over the 20-week study period for subjects who completed the study. PLA represented by *open square symbols*; PRO represented by *closed diamond symbols*. Data are mean and SEM. No difference between treatments. Significantly different from week 1: *p < 0.05, **p < 0.01

Analysis of the IPAQ questionnaires indicated that the training loads were not consistent over the 20 weeks of the study as illustrated in Fig. 1. The training loads decreased significantly in time (*p* = 0.001) from week 4 until week 10 after which the level returned to starting levels. In this ~20 % reduction in physical activity there was no statistical difference between the PRO and the PLA group (*p* = 0.709).

The period of reduced physical activity corresponded to the Christmas vacation and exam period during December–January.

Over the 20-week study period mean training loads were not different for the PRO and PLA groups: 79.4 (2.9) and 82.1 (3.2) MET-h/week, respectively (*p* = 0.545). This is equivalent to about 13 (1) hours of moderate-vigorous activity per week.

### URS incidence

**Fig. 2:**
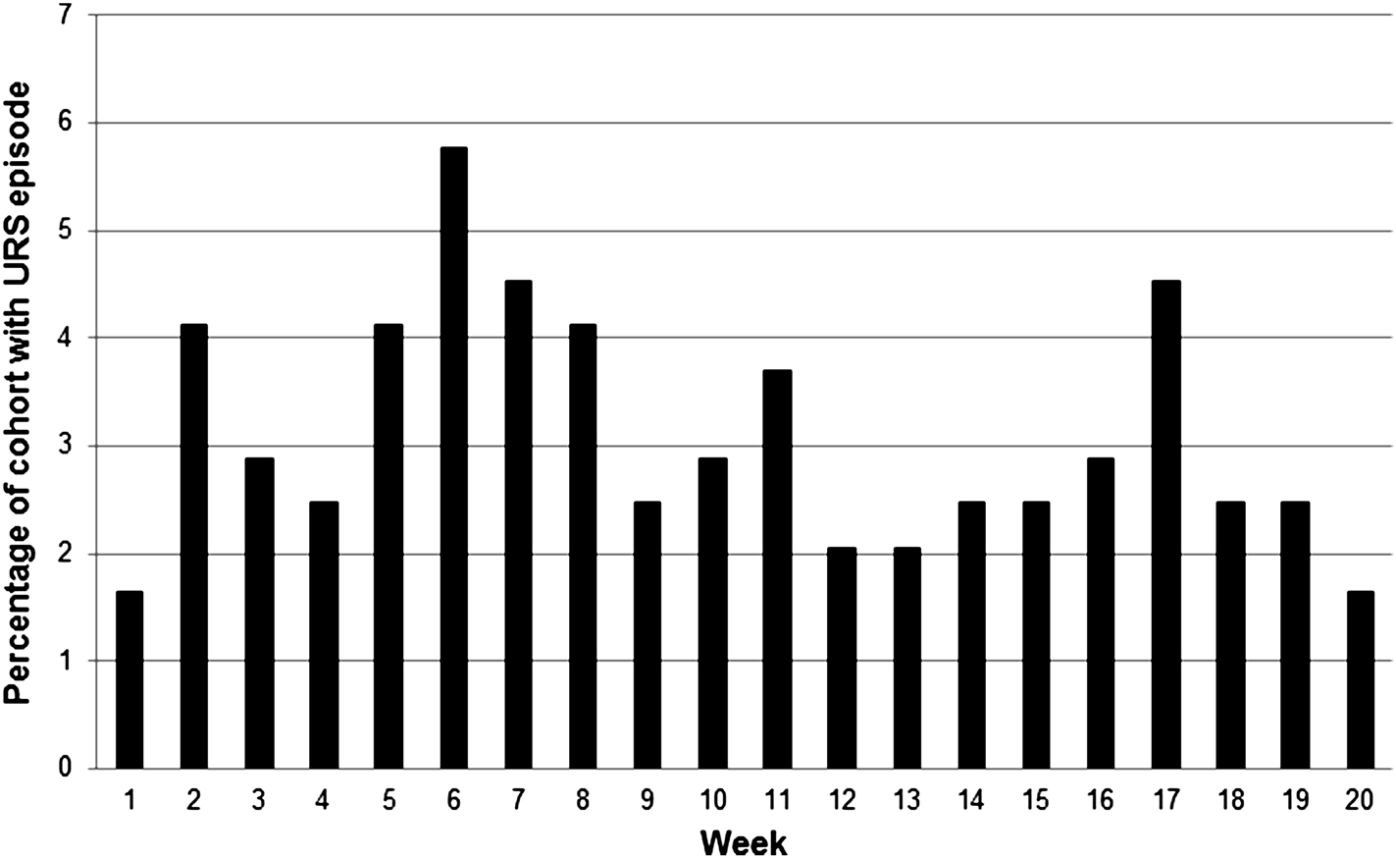
Percentage of the cohort reporting an URS episode for each week of the study period

133 subjects did not experience a single URS episode during the study period whereas 110 subjects experienced at least one URS episode during the study period. Within the whole subject cohort there was a rising incidence of infection during weeks 4–6, 9–11 and 13–17 (Fig. 2).

The proportion of subjects from the PLA group who experienced one or more URS episodes was 0.42 (i.e. 42 % of subjects in the group) whereas the proportion from the PRO group was 0.48. The difference in proportions was not significant (Chi squared test statistic = 0.820, *p* = 0.365). On average, subjects experienced their first URS episode in week 8 for both PRO and PLA groups.

The number of URS episodes, number of days with URS score ≥5 and mean total symptom score and duration of URS episodes were not different between PRO and PLA (Table 2). The proportion of subjects whose training was negatively affected when URS was present was not different between PRO and PLA (0.33 for both; Chi squared test statistic = 0.000, *p* = 1.000).

**Table 2:** Number of URS episodes, number of days with URS symptom score ≥5, and mean total symptom score and duration for URS episodes in the 20-week period (per person)

	PRO	PLA	*p*
URS episodes	0.7 ± 0.1	0.6 ± 0.1	0.417
# Days with score ≥5	8 ± 1	8 ± 1	0.404
Total symptom score	62 ± 5	64 ± 6	0.312
URS episode duration	5.6 ± 0.4	5.9 ± 0.5	0.713

Values are expressed as mean ± SEM

During weeks when an URS episode was present, the proportion of subjects who took medication was similar in the PRO and PLA groups (0.30 and 0.33, respectively; Chi squared test = 0.219, *p* = 0.640) and the proportion who went to see their doctor was also similar in the PRO and PLA groups (0.04 and 0.02, respectively; Chi squared test = 0.602, *p* = 0.438).

### Blood leukocyte counts

There were no significant differences either before or after the 20-week supplementation period between PLA and PRO in blood total or differential leukocyte counts (Table 3).

**Table 3:** Total and differential blood leukocyte counts (cells × 10^9^/L) before and after 20 weeks of the intervention period

	Before	20 weeks	*p* (PLA *vs* PRO: before, after 20 weeks)
Total Leukocytes			
PLA	6.17 ± 0.13	5.72 ± 0.11	0.401, 0.260
PRO	6.18 ± 0.13	5.85 ± 0.13	
Neutrophils			
PLA	3.30 ± 0.10	2.91 ± 0.11	0.789, 0.391
PRO	3.34 ± 0.12	3.06 ± 0.13	
Monocytes			
PLA	0.60 ± 0.02	0.58 ± 0.02	0.671, 0.997
PRO	0.61 ± 0.02	0.58 ± 0.01	
Lymphocytes			
PLA	1.98 ± 0.05	1.95 ± 0.05	0.134, 0.110
PRO	2.13 ± 0.05	2.06 ± 0.04	

Values are expressed as mean ± SEM

### Plasma herpesvirus antibody titres

**Fig. 3:**
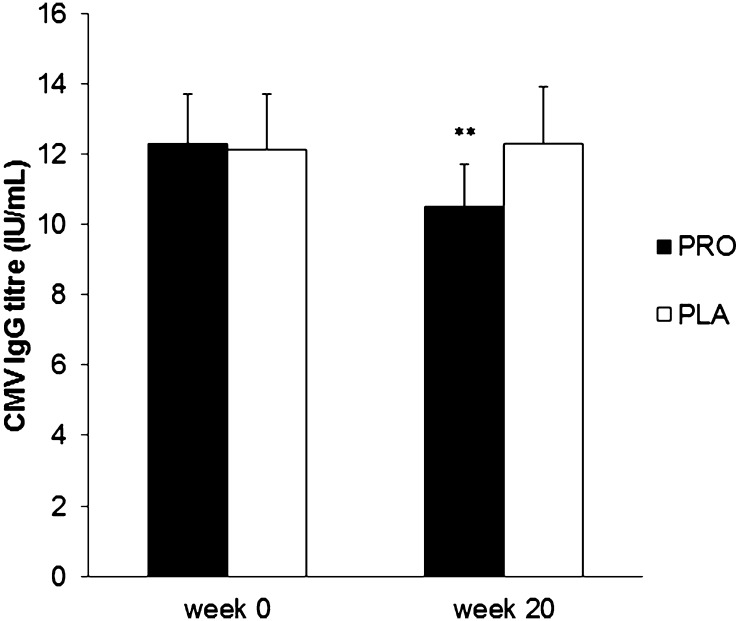
Plasma CMV IgG antibody titre in the PRO and PLA groups at the start and end of the study. Significant main effect of time (*p* = 0.023) and significant time × group interaction (*p* = 0.023). Significantly different from week 0 in PRO group only: **p < 0.01

In the PLA group 25 % of subjects were CMV seropositive at the start of the study and 23 % in the PRO group. At the end of the study a further 2 and 3 participants had become CMV seropositive in the PLA and PRO groups, respectively. A significant main effect of time (*p* = 0.023) and a significant time × group interaction effect (*p* = 0.003) was observed for plasma CMV IgG titres in CMV seropositive participants with antibody titre falling in the PRO group but remaining unchanged in the PLA group over time (Fig. 3).

**Fig. 4:**
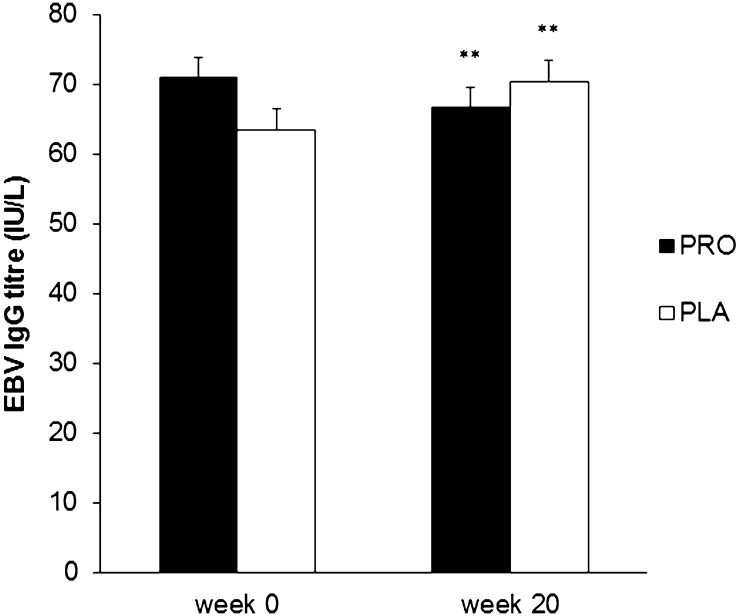
Plasma EBV IgG antibody titre in the PRO and PLA groups at the start and end of the study. Significant time × group interaction (*p* = 0.001). Significantly different from week 0: **p < 0.01 with decrease in PRO group and increase in PLA group

In the PLA group 67 % of subjects were EBV seropositive at the start of the study and 77 % in the PRO group. At the end of the study a further 6 and 3 participants had become EBV seropositive in the PLA and PRO groups, respectively. At the start of the study the EBV antibody titre was not significantly different in the PLA and PRO groups. A significant time × group interaction effect (*p* = 0.001) was observed for plasma EBV IgG titres in EBV seropositive participants with antibody titre falling in the PRO group but increasing in the PLA group over time (Fig. 4). Despite these significant interaction effects, herpesvirus antibody titres were not significantly different between PRO and PLA groups at the end of the study.

### Blinding

Of the 243 subjects who completed the study, 75 indicated that they had no idea which treatment they were on. Of the 168 subjects who expressed an opinion, 93 (55 %) were correct and 45 % were incorrect in their selection of treatment; hence, the study blinding was effective.

## Discussion

The present study investigated the effects of regular probiotic intake on self-reported URS in a group of highly active individuals engaged in their normal level of training and competition during the period October 2014 to March 2015. The main findings of the present study were that 20 weeks of daily probiotic supplementation did not reduce incidence of URS episodes or the number of days with a symptom score ≥5. There was also no difference between PRO and PLA in the proportion of subjects who experienced at least one URS episode. Similarly, in those who experienced one or more URS episodes, the duration and severity of the episodes were not different between PRO and PLA. However, regular ingestion of probiotic reduced the CMV and EBV antibody titres in seropositive participants.

The individuals recruited to this study were highly-trained, competitive athletes undertaking daily exercise and similar cohorts engaged in similar volumes of training have been shown to be at a higher risk of URS when exposed to pathogens (Gleeson et al. 2013a, b; Nieman et al. 1990). However, the mean number of URS episodes in the study cohort (0.6) was considerably lower than that expected based on the results of our previous smaller scale study (Gleeson et al. 2011) where it was 2.1 in the PLA group. The much lower rate of common cold incidence than anticipated among the athlete cohort examined in our present study may have prevented us from detecting a significant effect of probiotic supplementation on respiratory infection incidence. Essentially, our study was underpowered and the outcome is therefore not representative for the potential efficacy of the probiotic product. There are several possible reasons for the lower than expected URS incidence. Firstly, common cold and influenza incidence among the general population in the UK during the winter of 2014–2015 (Public Health England National Weekly Influenza Report 2015) was relatively low compared with the three previous years. Secondly, more guidelines for athletes on how to avoid infection are available in the public domain including review articles (e.g. Walsh et al. 2011b; Gleeson and Walsh 2012), websites (e.g. British Association of Sport and Exercise Sciences, English Institute of Sport), media, press and magazines (e.g. Runners’ World, Cycling Weekly). Thus, certainly in the UK, there is a diminishing population of illness prone athletes who are not aware of these countermeasures.

Despite the absence of any effect of probiotic on URS in the present study, there was a significant impact on overall immune status as indicated by the differences in the changes in the plasma CMV-specific and EBV-specific IgG titres on PRO and PLA from the start to the end of the study. The level of the CMV antibody in plasma of CMV seropositive individuals inversely reflects the overall immune system status and is part of the immune risk profile used to assess immune dysregulation in elderly people (Hadrup et al. 2006; Alonso Arias et al. 2013). Among young adult athletes in a previous study (He et al. 2013) 25 % of individuals were CMV seropositive and this was confirmed in the present study. Among elderly people the proportion of CMV seropositive individuals will be much higher [approx. 90 % in the over 60 s (Dowd et al. 2011)] and a recent double blind, placebo controlled study of elderly volunteers using a daily *Lactobacillus* probiotic for 6 months reported that the serum CMV IgG titre was significantly increased at the end of the study in the placebo group but was unchanged in the probiotic group possibly because their immune system was able to better control viral reactivations (Moro-García et al. 2013). In the present study *Lactobacilluscasei* Shirota ingestion significantly reduced the CMV antibody titre in CMV seropositive participants with no change in the placebo group. The percentage of individuals seropositive for EBV (72 %) was substantially higher than for CMV (24 %) in our cohort of student athletes as previously reported by He et al. (2013). In the present study, similar to the findings for CMV, *Lactobacilluscasei* Shirota ingestion significantly reduced the EBV antibody titre in EBV seropositive participants whereas there was a significant increase in the placebo group. These reductions in herpesvirus antibody titres with regular probiotic ingestion can be interpreted as indicating an overall benefit to immune system status. Such effects are desirable for long-term health as studies have shown that poor control of herpes virus reactivation may contribute to naïve T cell exhaustion and earlier onset of immunosenescence (Simpson et al. 2016). The present study does not provide any evidence of the mechanism(s) whereby herpesvirus antibody titres are reduced by the probiotic but modulation of host immunity is an important potential mechanism by which probiotics confer health benefits (Trejo and Sanz 2013). Studies by Dong et al. (2010, 2012) have established that *Lactobacillus casei* Shirota promotes natural killer cell activity, activates cytotoxic lymphocytes and increases T helper 1 cytokine production which could potentiate the destruction of virus-infected cells in the body, reducing viral replication and subsequent antiviral antibody production.

In summary, 20 weeks of daily *Lactobacilluscasei* Shirota probiotic supplementation did not reduce URS episode incidence, duration or severity in a group of highly active individuals engaged in their normal level of training and competition over the winter months. However, regular ingestion of this probiotic reduced plasma CMV and EBV antibody titres, an effect that can be interpreted as a benefit to overall immune status.

## Abbreviations

ANOVA: Analysis of variance
BMI: Body mass index
CMV: Cytomegalovirus
EBV: Epstein Barr
ELISA: Enzyme-linked immunosorbent assay
IgG: Immunoglobulin G
IPAQ: International Physical Activity Questionnaire
K3EDTA: Potassium ethylene diamine tetra acetic acid
PLA: Placebo
PRO: Probiotic
SD: Standard deviation
SEM: Standard error of mean
SIgA: Secretory immunoglobulin A
URS: Upper respiratory tract illness symptoms
